# Psychological perspectives on expertise

**DOI:** 10.3389/fpsyg.2015.00258

**Published:** 2015-03-10

**Authors:** Guillermo Campitelli, Michael H. Connors, Merim Bilalić, David Z. Hambrick

**Affiliations:** ^1^School of Psychology and Social Science, Edith Cowan UniversityJoondalup, WA, Australia; ^2^Department of Cognitive Science, ARC Centre of Excellence in Cognition and its Disorders, Macquarie UniversitySydney, NSW, Australia; ^3^Dementia Collaborative Research Centre, School of Psychiatry, University of New South WalesSydney, NSW, Australia; ^4^Department of General Psychology and Cognitive Science, Institute of Psychology, Alpen Adria University KlagenfurtKlagenfurt, Austria; ^5^Department of Psychology, Michigan State UniversityEast Lansing, MI, USA

**Keywords:** expertise, expert performance, expert cognitive processes, skill acquisition, skill transfer, training, deliberate practice

## Introduction

This Research Topic sought to advance psychological understanding of expertise by drawing together lines of research from many different domains of expertise. The outcome is a collection of 35 articles in such diverse areas as chess, music, perception, teaching, intensive-care diagnosis, video-games, sports, dance, mathematics, climbing, and fingerprint analysis.

The articles can be classed into five broad categories based on their focus: (a) the cognitive processes in expertise, (b) the development of expertise, (c) the relationship between expertise and general cognition, (d) the transfer of skills between domains, and (e) methodological issues and frameworks in expertise research. We give a brief overview of the research across these five themes.

## Cognitive processes in expertise

Articles in this research topic used a number of different methodologies to investigate cognitive processes in expertise. Four articles examined experts' eye movements as a way of studying what experts focus on when performing domain-relevant tasks. Sheridan and Reingold (2014), for example, found that expert chess players rapidly differentiate regions of the board that are relevant to the best move from irrelevant ones. Similarly, McCormack et al. (2014) found that expert intensive-care physicians directed their attention to more relevant areas of the situation, compared to competent non-experts. In addition, Godau et al. (2014) found that experts in arithmetic problem solving spontaneously used arithmetic shortcuts. Finally, Ellis and Reingold (2014) examined the Einstellung effect (i.e., where the first idea that comes to mind blocks finding the best solution to a problem) using this methodology and noted its relevance to understanding expert flexibility (see Bilalić and McLeod, 2014).

Two articles focused on perceptual expertise. Curby and Gauthier (2014) found that acquiring expertise with a category of stimuli (i.e., car expertise) increases the interference between the visual processing of other familiar stimuli (e.g., faces) and that of the learned category (cars). In a study with novel objects, Cheung and Gauthier (2014) found that acquiring perceptual expertise involves integrating perceptual and conceptual representations of stimuli.

Four studies investigated expertise involving physical movements. In a study on dancing, Bläsing (2015) found that making a sequence of movements influenced the subsequent perception of that sequence, but not to the same degree if one was a dancing expert. In a study of climbing experts, Bläsing et al. (2014) found that expertise was associated with better perception of climbing holds and action-relevant objects. In a study of athletes and musicians, Braun Janzen et al. (2014) showed that training affects performance involving timing and rhythmic movements: athletes were more precise making continuous movements, whereas musicians were more precise for discrete movements. In a study of skill acquisition in a flight simulator, Wiggins et al. (2014) found that a general capacity for acquiring and using cues was related to performance in landing an aircraft in the simulator.

Finally, four articles focused on experts' pattern recognition (the ability to identify meaningful relationships in complex stimuli). In a review of research on fingerprint experts, Thompson et al. (2014) concluded that such expertise relies on rapid pattern recognition and discrimination rather than in analytic thinking. In an observational study, Kretz and Krawczyk (2014) found that academic economists use many analogies in research meetings. Trench (2014), however, suggests that these results may be due to the naturalistic setting of the study, rather than expertise *per se*. Bialek and Sawicki (2014) showed that participants asked to take an expert perspective become more risk aversive and patient in decision making tasks. Finally, Leone et al. (2014) examined the relationship between expertise and representations of space using a large dataset of chess games from an internet server. They found that novices, relative to experts, use strategies to reduce their cognitive load (see Connors and Campitelli, 2014, for a commentary).

## Development of expertise

Six studies examined how expertise is developed. Gaschler et al. (2014) examined the learning curves in skill acquisition by analyzing the tournament performance of 1383 chess players over 10 years. They found that exponential learning curves better fitted players' improvements over time than power function learning curves. Gobet and Ereku (2014) discussed the case of Magnus Carlsen, current world chess champion, and argue that his level of performance cannot be accounted for by the deliberate practice account, which suggests that amount of deliberate practice is the critical determinant of expertise.

Citing limitations in an earlier meta-analysis by Hambrick et al. (2014a), Platz et al. (2014) conducted a meta-analysis on the influence of deliberate practice in musical achievement. They found a moderate average effect size (*r_c_* = 0.61), which they interpret as showing the importance of deliberate practice. In response to Platz et al.'s criticisms, Hambrick et al. (2014b) noted a number of conceptual problems in Platz et al.'s arguments and observe how Platz et al.'s findings can also be interpreted to show that practice, while undoubtedly an important factor in expertise, is not the sole determinant.

Healy et al. (2014) proposed a number of training principles for developing expertise. These include the acquisition of expertise (e.g., scheduling of feedback), retention of expertise (e.g., item chunking, depth of processing) and transfer (e.g., variability of practice, seeding the knowledge base). Finally, Speelman (2014) argued that treating numeracy as a form of expertise and using computer programs in teaching would address some shortcomings in current teaching and, in particular, foster a greater focus on practice and feedback in learning.

## Expertise and general cognition

Three articles examined the relationship between expertise and general cognition. First, Gobet et al. (2014b) discussed how artificial intelligence and engineering could be used to design a brain. Based on expertise research, they propose that a better brain would have less concepts and more low-level perceptual processing. Second, Guida and Lavielle-Guida (2014) combined findings from memory research with the normal population with theories of expert memory. They argue that a less sophisticated version of the spatial method of loci used by memory experts is also used by ordinary people to encode items in working memory. Third, Christophel et al. (2014) observed that amount of teaching experience is a very poor predictor of a teacher's actual effectiveness, including, for example, the teacher's ability to offer constructive feedback to students.

## Transfer of skills

Three articles examined the transfer of skills across domains. First, Gobet et al. (2014a) investigated the possibility of training transfer from videogame playing to selective attention and working memory capacity. Consistent with over a century of research, there was no evidence for transfer, even in videogame experts. In contrast, Lampit et al. (2014) reported evidence for transfer of computerized cognitive training to a bookkeeping task. Finally, Bart (2014) reviewed research published after Gobet and Campitelli's (2006) critical review on the effects of chess education, showing statistically significant effects of chess education on academic achievement.

## Frameworks, recommendations, and methodology

Seven articles discussed expertise in general. First, Vaci et al. (2014) consider alternative approaches to studying expertise, and in particular, how studying only individuals from highly restricted ranges of skill may yield different findings than studying individuals who represent wider ranges of skill. Second, Kaufman (2014) identifies points of disagreement and agreement in different views of expertise and suggests some directions for future research. Third, Bourne et al. (2014), categorize expertise as elite, peak, or exceptionally high levels of performance on a particular task or within a given domain.

Fourth, Shen et al. (2014) use birdwatching as an illustrative example to discuss such issues as selecting an appropriate domain of perceptual expertise for study, recruiting experts, assessing their level of expertise, and experimentally testing the experts' performance. Fifth, MacIntyre et al. (2014) propose that athletes are not just experts in movement execution but also in planning, metacognition, and reflection. Similarly, Toner and Moran (2014), extending Sutton et al.'s (2011) framework, argue that expert athletes do not completely automatize their skills and that an important component of their expertise is to be able to rapidly reflect on their movements. Finally, de Oliveira et al. (2014) build upon Gigerenzer's (e.g., Gigerenzer and Goldstein, 1999) heuristic-based approach to decision making. They propose that expert athletes develop a toolbox of heuristics to guide their decision making.

## Conclusion

The diversity of articles in this research topic illustrates the many different approaches to studying expertise. It also indicates the keen interest in the topic. We believe that many articles in this research topic are of lasting importance and can help to guide future research in the field of expertise.

### Conflict of interest statement

The authors declare that the research was conducted in the absence of any commercial or financial relationships that could be construed as a potential conflict of interest.
